# Predicting adherence to fully-automated, chatbot-delivered digital cognitive behavioral therapy for insomnia (dCBT-I) using machine learning: A pilot real-world study

**DOI:** 10.1371/journal.pdig.0001170

**Published:** 2026-01-02

**Authors:** Rose Wing Lai So, Kit Ying Chan, Christopher Chi Wai Cheng, Ngan Yin Chan, Shirley Xin Li, Joey Wing Yan Chan, Steven Wai Ho Chau, Yun Kwok Wing, Tim Man Ho Li

**Affiliations:** 1 Li Chiu Kong Family Sleep Assessment Unit, Department of Psychiatry, The Chinese University of Hong Kong, Hong Kong, China; 2 Department of Psychology, The University of Hong Kong, Hong Kong, China; 3 The State Key Laboratory of Brain and Cognitive Sciences, The University of Hong Kong, Hong Kong, China; 4 Li Ka Shing Institute of Health Sciences, Faculty of Medicine, The Chinese University of Hong Kong, Hong Kong, China; Henry Ford Health System, UNITED STATES OF AMERICA

## Abstract

Digital cognitive behavioral therapy for insomnia (dCBT-I) is effective in treating insomnia, but adherence remains a major challenge in real-world applications. Machine learning (ML) offers potential in predicting healthcare utilization. This study applied ML techniques to predict adherence to dCBT-I based on participant baseline characteristics. This pilot real-world study included 75 individuals (69% female; 41% aged 35–44 years) with insomnia symptoms (Insomnia Severity Index, ISI ≥ 8) who participated in a 28-day chatbot-delivered dCBT-I program. ML models, including logistic regression with elastic-net penalty, support vector machine, random forest, and gradient boosting, analyzed participant baseline characteristics to predict adherence to dCBT-I in terms of session completion, usage duration, and response volume. These models were fine-tuned using grid search and evaluated with cross-validation. The synthetic minority over-sampling technique was applied to address data imbalances in the training set. Baseline depressive symptoms were the most predictive of non-adherence. Higher depressive symptoms were associated with shorter overall usage duration (β = -3.57, 95% CI: -5.82 to -1.33, p = .002). Longer sleep onset latency and wake time after sleep onset from the previous night increased the number of responses and longer usage duration on the following day (β = 0.01-0.05, p < .05). No significant associations were found between daytime and bedtime usage and sleep parameters for that specific night. ML models predicted overall adherence, with AUCs of 0.65-0.91 (p < .05). ML models also predicted next-day adherence, with AUCs of 0.56-0.74 (p < .05). This real-world study demonstrates the potential of ML to predict user adherence to dCBT-I and provides clinical insights for personalizing sleep-focused treatments. The study also investigated daily usage and adherence patterns in dCBT-I to predict next-day adherence.

## Author summary

Digital cognitive behavioral therapy for insomnia (dCBT-I) is an effective treatment for insomnia, yet many people find it difficult to consistently follow the program in real-world settings. Our research shows that machine learning can help identify individuals who are likely to struggle with adherence, enabling therapists to provide targeted support for them. There is limited knowledge about what predicts adherence in the context of dCBT-I, but we discovered that baseline depressive symptoms are a strong indicator of non-adherence. In addition, no prior research has examined how daily dCBT-I usage relates to next-day adherence. Our findings revealed that longer sleep onset latency and wake time after sleep onset from the previous night led to increased responses and usage the following day. While the use of digital devices emitting blue light before bedtime is often thought to negatively impact sleep, our study reveals that nighttime use of the dCBT-I intervention did not correlate with poor sleep on that night.

## Introduction

Insomnia is a prevalent sleep disorder, affecting 5.8-32.8% of people worldwide [1]. Cognitive behavioral therapy for insomnia (CBT-I) is the first-line treatment for chronic insomnia [2]. However, in-person delivery faces challenges such as clinician shortages, limited accessibility, high costs, and stigma associated with help-seeking. To address these barriers, the digital and automated forms of CBT-I have become more widely adopted. Recent meta-analyses have demonstrated that digital CBT-I (dCBT-I) effectively reduces insomnia [3,4].

Digital interventions are found more effective when provided with higher interactivity [5,6]. Chatbots are increasingly utilized in mental health services, transforming digital interventions from one-way informational content to interactive, patient-centered conversational agents. Mental health chatbots, such as Wysa, Woebot, Youper, and Tess, offer feedback and encourage users to complete interventions [7–10]. Users have reported positive experiences with chatbots, noting high acceptability, usefulness, and ease of use, along with a greater willingness to self-disclose, compared to human operators [11,12]. To fully automate dCBT-I, there are emerging studies employing chatbots as a delivery method. For instance, a study has shown that the Sleep Ninja chatbot improved insomnia, sleep quality, and depression among adolescents [13]. A randomized controlled trial involving 82 participants has also revealed that a dCBT-I chatbot significantly reduced insomnia symptoms and improved sleep duration and efficiency compared to a sleep education app [14].

Digital interventions, however, face significant challenges, particularly low adherence, as evidenced by a systematic review showing that only 0.5-28.6% of users would engage with all modules in real-world digital self-help interventions [15]. Adherence measures, including tracking completed online modules/levels/activities, treatment duration, and consumed materials, have been employed in studies on digital and self-help interventions [16]. Predicting user adherence is essential for enhancing treatment delivery and healthcare utilization, with predictors including demographic variables, sleep-related questionnaires, diary-derived sleep variables, health and psychological variables, and attitudes toward treatment [16–18]. Nevertheless, only a limited number of CBT-I studies (6 out of 102) specifically investigated adherence predictors in the context of dCBT-I [16]. Furthermore, no research has been conducted to examine daily usage and adherence patterns in dCBT-I concerning next-day adherence.

Machine learning (ML) presents a promising approach for predicting healthcare utilization [19]. Unlike traditional statistical models that explore variable relationships based on mathematical assumptions, ML models focus on learning patterns and relationships directly from the data. ML models prioritize predictive accuracy by identifying patterns and trends in the data without relying on predefined relationships or assumptions. This study employs the ML methodology to predict adherence to dCBT-I in terms of session completion, usage duration, and response volume. Specifically, this real-world intervention study aims to (a) examine participant baseline characteristics associated with overall and daily adherence to dCBT-I and (b) utilize the characteristics as input features to evaluate the performance of ML models in predicting overall and next-day adherence in the real-world settings.

## Methods

### Study design

This pilot feasibility research employed a real-world study design. We launched an intervention service which was promoted to local communities through a multifaceted outreach campaign. This included social media advertisements, booth exhibition engagements, and institutional mass emails. The promotional efforts ran from April 2023 to May 2024 in Hong Kong. Members of the public could access the service using the promotion link. The intervention service and questionnaire administration were automated and conducted through online platforms (S1 Fig), without any direct interaction between researchers and participants. After completing baseline questionnaires, service users were able to access a free 28-day chatbot-delivered dCBT-I program. Prior to completing the questionnaires, users could only access the basic, limited features of the program.

To be included in the study, users had to meet the following criteria: (i) aged 18–65 years, (ii) able to understand Cantonese, (iii) provided online informed consent, (iv) willing to comply with the requirements of the study protocol, (v) had insomnia measured by a score of 8 or above in the Insomnia Severity Index (ISI), (vi) had access to a smartphone and consistent internet access for their smartphone, (vii) accepted the terms of service and privacy policies of the instant messaging app used in the study for the delivery of the intervention. Users who reported a history of psychiatric disorder(s) were excluded from the analysis. During the study period, the intervention had a total of 347 users. Of these, 27% (n = 94/347) completed the baseline questionnaire, and 80% (n = 75/94) of those respondents were identified as having insomnia (ISI ≥ 8). Participants did not receive any incentives.

### Ethics statement

Ethics approval was obtained from the Joint Chinese University of Hong Kong – New Territories East Cluster clinical research ethics committee (reference no: 2024.078). Participants provided online informed consent.

### Intervention

The intervention employed a self-developed chatbot named “Master Sleep” to deliver all components of CBT-I in the local colloquial Cantonese language. The program was structured according to the four key modules of standard CBT-I [20]: (Day 1–7) psychoeducation about sleep and insomnia, (Day 8–14) sleep hygiene education and behavioral strategies (including stimulus control and sleep restriction), (Day 15–21) cognitive restructuring and constructive worry strategies, and (Day 22–28) stress management and relaxation training. Participants progressed through the modules sequentially, and a new module was released only after they had completed the content in the previous module. Participants interacted with the chatbot through an instant messaging format on the Telegram platform. Participants engaged in the chatbot through free-text input or by responding to questions with preselected options. The chatbot’s replies were drawn from a predetermined set of standardized statements and follow-up questions.

Each day, the chatbot initiated conversations based on predefined rules, guiding participants through a structured 28-day program. The process began with participants completing a daily sleep diary, where they recorded their sleep-wake patterns, including total sleep time (TST), total time spent in bed (TIB), wake time after sleep onset (WASO), and sleep onset latency (SOL). The chatbot automatically calculated participants’ sleep efficiency (SE) from this data (S2 Fig) and discussed appropriate bedtimes and wake-up times regularly as part of the sleep restriction component throughout the intervention. After submitting their sleep diary and upon completing the previous day’s sessions, participants received a new intervention session each day. The intervention was delivered through a combination of text, graphics, and audio (S3 Fig). Users could complete both the unfinished sessions and the new session on the same day if they had not completed the previous sessions.

Following the review of each daily session, participants could ask questions at any time. The chatbot facilitated the Q&A feature, allowing users to inquire about the intervention materials (see S4 Fig). Using machine classification techniques [21–23], the chatbot provided relevant responses to address participants’ questions and concerns. In addition, it identified the sentiment expressed in participants’ messages, enabling it to offer appropriate responses, such as guiding the participant through relaxation techniques or providing constructive worry strategies. If severe distress was detected, the research team would reach out to at-risk participants for follow-up. However, there were no such cases in the current study.

### Measures

User behaviors were automatically tracked each day throughout the intervention, capturing data such as the number of sessions completed, total usage duration in minutes, and the number of text messages sent by participants to the chatbot, both overall and per day. Overall adherence measures derived from user behavior included the total number of sessions completed, the cumulative usage duration, and the total count of user responses throughout the intervention. Daily adherence metrics consisted of the daily counts of completed sessions, usage duration, and user responses. Good adherence was defined as exceeding the average values by at least one standard deviation (SD).

Predictors of adherence included baseline characteristics of participants, specifically demographic variables (age and gender), diary-derived sleep variables (TST, TIB, WASO, SOL, and SE), user behaviors on the first day of the intervention (total number of responses, total usage duration, average response time, and the SD of response time) and responses to sleep-related and depression questionnaires. For predicting next-day adherence, diary-derived sleep variables from the previous night were also included.

Five sleep-related and depression questionnaires were administered at baseline. First, the ISI assessed the severity of insomnia symptoms using 7 items on a 5-point Likert scale, where a score of 8 or above indicated insomnia. Second, the reduced Horne and Östberg Morningness and Eveningness Questionnaire (rMEQ) assessed chronotype preference through 5 items, with the first four scored from 1 to 5 and the last from 0 to 6, resulting in a total score range of 4–26. Lower scores indicated a preference for eveningness. Third, the Dysfunctional Beliefs and Attitudes about Sleep Scale (DBAS) included 16 items on an 11-point Likert scale, evaluating faulty sleep-related beliefs and cognitions. Fourth, the Sleep Hygiene Index (SHI) comprised 13 items on a 5-point Likert scale to assess sleep hygiene behaviors. Fifth, the Patient Health Questionnaire-9 (PHQ-9) utilized 9 items on a 4-point Likert scale to measure the severity of depressive symptoms.

### Statistical analysis

All analyses were conducted using R (version 4.4.1) and Python (version 3.11.9). Statistical significance was set as p < .05. Categorical variables (age, gender) were presented as numbers and percentages, while means and SDs summarized baseline measures, sleep parameters, and user behaviors throughout the intervention. Baseline self-report measures, sleep parameters, and first-day user behaviors were compared between those with good adherence and other participants using one-way analysis of variance, with age and gender compared using the Chi-square test.

#### Associations of user behaviors with participant characteristics.

A multiple linear regression model was used to explore the relationships between baseline characteristics of participants (significant independent variables from simple linear regression models) and overall user behaviors throughout the intervention (the dependent variable), with regression coefficients and 95% confidence intervals reported. Furthermore, multiple linear mixed-effects modeling was utilized to explore the relationships between daily user behaviors and sleep parameters, with participant ID as a random effect. The analysis was adjusted for age, gender, and the sequence of sleep diary entries. Sleep parameters from the previous night were used as predictors for user behaviors on the following day. When no user behaviors were recorded the next day, these instances were marked as 0. Daytime and nighttime user behaviors also served as predictors for sleep parameters at night. Nighttime intervention usage was defined as user behaviors occurring within 2 hours before bedtime. If sleep parameters for a specific night were missing, these data points were treated as missing.

#### Prediction of overall and daily adherence to the intervention.

Four machine learning models were employed to identify participants with good adherence: logistic regression (LR) with elastic-net penalty (representing the linear approach), support vector machine (SVM; kernel-based approach), and random forest (RF) and gradient boosting (GB; tree-based approaches). To predict overall adherence, baseline characteristics of participants were used as input features. Diary-derived sleep variables from the previous night were also included to predict next-day adherence. Models were refined through grid search with nested cross-validation for optimal hyperparameter tuning. To address data imbalance, the synthetic minority over-sampling technique was implemented. For overall adherence with a small sample size, leave-one-out cross-validation was employed to maximize the use of available data for training and testing. For daily adherence with more data points, stratified 5-fold cross-validation was used to provide an unbiased estimation of performance. The performance of the models was evaluated using Receiver Operating Characteristic curve analysis, which included metrics such as the area under the curve (AUC), 95% confidence intervals, sensitivity, and specificity. SHapley Additive exPlanations (SHAP) was utilized to interpret the predictions of the best models.

#### Diary-derived sleep variables of participants with good overall adherence.

An intent-to-treat, mixed-effects model for repeated measures was used to assess differences in daily sleep parameters (TST, TIB, WASO, SOL, SE) between the adherence and non-adherence groups over time. Participant ID was set as a random effect, and all analyses were adjusted for age and gender.

## Results

### Overall user behaviors and adherence

#### Associations of overall user behaviors with participant baseline characteristics.

On average, participants completed 10.80 ± 10.31 sessions, spent 51.25 ± 59.55 minutes, and provided 263.37 ± 268.35 responses throughout the intervention, with a mean interval of 4.37 ± 8.10 days between sessions. Higher baseline PHQ-9 scores predicted shorter usage duration (β = -3.57, 95% CI: -5.82 to -1.33, p = .002), while longer usage duration on the first day predicted greater overall usage duration (β = 2.95, 95% CI: 1.13 to 4.77, p = .002) and daily usage duration (β = 0.39, 95% CI: 0.23 to 0.55, p < .001). Higher baseline ISI (β = 0.94, 95% CI: 0.12 to 1.76, p = .03) and more responses on the first day (β = 0.18, 95% CI: 0.02 to 0.33, p = .03) predicted a higher number of daily responses.

#### Baseline characteristics of participants with good overall adherence.

Among the 75 participants, the majority were female (69%, n = 52/75) and aged 35–44 years (41%, n = 31/75). Table 1 summarizes baseline characteristics of participants with good adherence. Those with good adherence in session completion (23%, n = 17/75) had lower PHQ-9 scores, shorter TIB, and more responses on the first day. Those with good adherence in usage duration (16%, n = 12/75) showed lower ISI and PHQ scores, and more responses and usage duration on the first day. Participants with good adherence in response volume (16%, n = 12/75) had lower ISI and PHQ scores, and more responses on the first day.

**Table 1:** Baseline characteristics of participants with good adherence.

	Session completion			Usage duration				Response volume		
	Others (n = 58)	Good adherence (n = 17)	p	Others (n = 63)	Good adherence (n = 12)	p	Others (n = 63)		Good adherence (n = 12)	p
Gender	n (%)	n (%)	.31	n (%)	n (%)	.14	n (%)		n (%)	.42
Male	20 (34)	3 (18)		22 (35)	1 (8)		21 (33)		2 (17)	
Female	38 (66)	14 (82)		41 (65)	11 (92)		42 (67)		10 (83)	
Age			.71			.23				.72
18-24	3 (5)	0 (0)		3 (5)	0 (0)		3 (5)		0 (0)	
25-34	15 (26)	5 (29)		19 (30)	1 (8)		18 (29)		2 (17)	
35-44	25 (43)	6 (35)		26 (41)	5 (42)		26 (41)		5 (42)	
45-54	10 (17)	3 (18)		10 (16)	3 (25)		10 (16)		3 (25)	
55-65	5 (9)	3 (18)		5 (8)	3 (25)		6 (10)		2 (17)	
Baseline self-report measures	Mean (SD)	Mean (SD)		Mean (SD)	Mean (SD)		Mean (SD)		Mean (SD)	
ISI	16.52 (3.92)	14.53 (4.23)	.08	16.57 (3.92)	13.42 (3.82)	.01	16.56 (3.93)		13.50 (3.87)	.02
rMEQ	11.21 (3.87)	12.47 (3.16)	.22	11.27 (3.83)	12.67 (3.06)	.24	11.44 (3.86)		11.75 (3.14)	.80
PHQ-9	13.31 (5.50)	9.88 (5.18)	.03	13.41 (5.47)	7.92 (3.68)	.001	13.25 (5.36)		8.75 (5.43)	.01
DBAS	103.47 (23.11)	109.29 (17.09)	.34	104.03 (22.57)	108.75 (18.47)	.50	104.06 (22.55)		108.58 (18.65)	.52
SHI	27.38 (6.13)	29.82 (5.65)	.15	27.65 (6.14)	29.42 (5.74)	.36	27.62 (6.07)		29.58 (6.08)	.31
First sleep diary entry										
SOL (mins)	37.43 (51.26)	38.53 (47.29)	.94	38.35 (51.80)	34.17 (41.66)	.79	38.35 (51.87)		34.17 (41.17)	.79
WASO (mins)	27.22 (32.62)	26.76 (34.37)	.96	27.25 (31.73)	26.42 (39.43)	.94	29.35 (34.05)		15.42 (22.81)	.18
TIB (mins)	534.84 (147.49)	457.65 (94.06)	.046	524.30 (141.24)	480.83 (136.18)	.33	529.62 (144.44)		452.92 (98.64)	.08
TST (mins)	433.67 (159.95)	373.24 (97.08)	.14	426.67 (157.20)	384.83 (98.82)	.38	427.51 (157.78)		380.42 (91.07)	.32
SE (%)	79.83 (16.78)	81.94 (16.63)	.65	80.14 (17.37)	81.17 (12.83)	.85	79.62 (17.43)		83.92 (11.71)	.42
First-day user behaviors										
Total number of responses	41.86 (32.42)	59.65 (32.57)	.05	42.08 (32.28)	65.92 (31.23)	.02	42.75 (31.72)		62.42 (36.62)	.06
Total usage duration (mins)	8.58 (7.27)	11.22 (5.63)	.17	8.17 (6.69)	14.47 (6.30)	.004	8.87 (7.27)		10.77 (5.24)	.39
Average response time (mins)	0.21 (0.11)	0.21 (0.11)	.90	0.20 (0.11)	0.24 (0.09)	.29	0.21 (0.11)		0.19 (0.07)	.66
SD of response time (mins)	0.29 (0.32)	0.29 (0.31)	.98	0.29 (0.34)	0.27 (0.16)	.87	0.30 (0.34)		0.23 (0.17)	.48

ISI: Insomnia Severity Index; rMEQ: Reduced Horne and Östberg Morningness and Eveningness Questionnaire; PHQ-9: Patient Health Questionnaire-9; DBAS: Dysfunctional Beliefs and Attitudes about Sleep; SHI: Sleep Hygiene Index scores; SOL: Sleep onset latency; WASO: Wake time after sleep onset; TIB: Total time spent in bed; TST: Total sleep time; SE: Sleep efficiency.

#### Prediction of overall adherence to the intervention.

Good overall adherence was defined as completing at least 21 sessions (75% of total), using the chatbot at least 110.80 minutes, or providing 531 responses throughout the intervention. As shown in Table 2, the LR (AUC = 0.70, p = .009), SVM (AUC = 0.65, p = .02), and GB (AUC = 0.73, p < .001) models significantly predicted good adherence in session completion. The LR (AUC = 0.74, p = .03), RF (AUC = 0.83, p < .001), and GB (AUC = 0.91, p < .001) models predicted good adherence in usage duration. The LR (AUC = 0.73, p = .007) and GB (AUC = 0.77, p < .001) models predicted good adherence in response volume.

**Table 2:** Prediction of overall adherence to the intervention using machine learning with participant baseline characteristics with leave-one-out cross-validation.

Machine learning techniques	AUC (95% CI)	p	Sensitivity (%)	Specificity (%)
Good overall adherence in session completion (23%, n = 17/75)				
LR	0.70 (0.55, 0.84) **	.008	58.82	60.34
RF	0.61 (0.46, 0.77)	.16	58.82	58.62
SVM	0.65 (0.52, 0.79) *	.02	58.82	58.62
GB	0.73 (0.63, 0.81) ***	<.001	68.97	67.24
Good overall adherence in usage duration (16%, n = 12/75)				
LR	0.74 (0.53, 0.94) *	.03	66.67	69.84
RF	0.83 (0.74, 0.92) ***	<.001	75.00	74.60
SVM	0.69 (0.49, 0.88)	.06	66.67	66.67
GB	0.91 (0.86, 0.96) ***	<.001	88.89	77.78
Good overall adherence in response volume (16%, n = 12/75)				
LR	0.73 (0.56, 0.90) **	.007	58.33	60.32
RF	0.65 (0.45, 0.84)	.14	66.67	66.67
SVM	0.68 (0.47, 0.89)	.10	58.33	58.73
GB	0.77 (0.68, 0.85) ***	<.001	68.25	73.02

* < .05, ** < .01, *** < .001; LR: Logistic Regression with elastic-net penalty; SVM: Support Vector Machine; RF: Random Forest; GB: Gradient Boosting.

**Fig 1:**
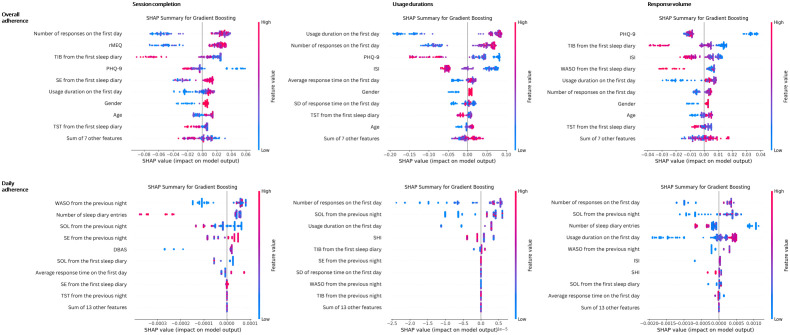
SHapley Additive exPlanations (SHAP) summary for gradient boosting models predicting overall and daily adherence. ISI: Insomnia Severity Index; rMEQ: Reduced Horne and Östberg Morningness and Eveningness Questionnaire; PHQ-9: Patient Health Questionnaire-9; DBAS: Dysfunctional Beliefs and Attitudes about Sleep; SHI: Sleep Hygiene Index scores; SOL: Sleep onset latency; WASO: Wake time after sleep onset; TIB: Total time spent in bed; TST: Total sleep time; SE: Sleep efficiency.

Fig 1 presents SHAP summary plots for the gradient boosting models used to predict overall adherence. The most influential predictors across all adherence outcomes were the number of responses and usage duration on the first day, PHQ-9 score, TST from the initial sleep diary entry, gender, and age. Specifically, a greater number of responses and longer usage duration on the first day, lower baseline TST and PHQ-9 scores, older age, and female gender were associated with higher predicted adherence, as indicated by positive SHAP values.

#### Diary-derived sleep variables of participants with good overall adherence.

Participants with good adherence in session completion (β = 0.15, 95% CI: 0.01 to 0.29, p = .03) and usage duration (β = 0.13, 95% CI: 0.01 to 0.25, p = .03) experienced increased SE over the intervention (see Table 3). Significant time x group interactions for WASO were found for both session completion (β = -0.60, 95% CI: -0.98 to -0.21, p = .002) and usage duration (β = -0.44, 95% CI: -0.80 to -0.08, p = .02). Post-hoc analyses revealed that those with good adherence in session completion (β = -0.36, 95% CI: -0.55 to -0.18, p < .001) and usage duration (β = -0.37, 95% CI: -0.59 to -0.16, p = .001) showed significant improvements in WASO compared to those without good adherence.

**Table 3:** Diary-derived sleep variables of participants with good overall adherence throughout the intervention.

Sleep parameters (DV)	Time effect		Group effect		Time x group effect	p
	β (95% CI)	p	β (95% CI)	p	β (95% CI)	
Session completion						
SOL	-0.29 (-0.66, 0.08)	.13	-8.35 (-28.34, 11.64)	.42	0.34 (-0.09, 0.78)	.12
WASO	0.24 (-0.09, 0.56)	.15	-2.87 (-14.88, 9.14)	.65	-0.60 (-0.98, -0.21) **	.002
TIB	-0.30 (-1.94, 1.31)	.72	-73.07 (-121.82, -24.28) **	.006	0.75 (-1.15, 2.71)	.45
TST	0.79 (-0.82, 2.38)	.33	-41.42 (-95.49, 12.68)	.15	-0.14 (-2.02, 1.76)	.89
SE	0.15 (0.01, 0.29) *	.03	3.58 (-2.56, 9.72)	.27	-0.11 (-0.27, 0.05)	.19
Usage duration						
SOL	-0.27 (-0.59, 0.05)	.10	-14.77 (-38.39, 8.85)	.23	0.37 (-0.03, 0.77)	.08
WASO	0.06 (-0.22, 0.34)	.66	6.19 (-8.12, 20.50)	.41	-0.44 (-0.80, -0.08) *	.02
TIB	-0.46 (-1.90, 0.96)	.53	-37.95 (-98.65, 22.83)	.24	0.89 (-0.91, 2.71)	.34
TST	0.58 (-0.82, 1.97)	.42	-28.24 (-92.88, 36.43)	.40	0.09 (-1.68, 1.85)	.92
SE	0.13 (0.01, 0.25) *	.03	2.57 (-4.73, 9.87)	.50	-0.09 (-0.24, 0.06)	.26
Response volume						
SOL	-0.20 (-0.62, 0.22)	.35	-13.93 (-36.58, 8.72)	.24	0.22 (-0.26, 0.69)	.37
WASO	-0.15 (-0.52, 0.22)	.44	-5.20 (-18.73, 8.33)	.46	-0.06 (-0.48, 0.36)	.77
TIB	-0.91 (-2.76, 0.93)	.33	-60.46 (-117.36, -3.50) *	.05	1.44 (-0.68, 3.53)	.18
TST	0.42 (-1.38, 2.24)	.65	-31.91 (-93.49, 29.71)	.32	0.32 (-1.76, 2.36)	.76
SE	0.12 (-0.04, 0.27)	.14	3.74 (-3.22, 10.69)	.30	-0.06 (-0.23, 0.11)	.51

* < .05, ** < .01, *** < .001; SOL: Sleep onset latency; WASO: Wake time after sleep onset; TIB: Total time spent in bed; TST: Total sleep time; SE: Sleep efficiency.

### Daily user behaviors and adherence

#### Associations of daily user behaviors with participant characteristics.

Based on 972 daily records, participants averaged 0.70 ± 1.03 sessions, 4.52 ± 3.67 minutes, and 24.85 ± 15.42 responses per day. At nighttime (2 hours before bedtime), they utilized the intervention 1.97 times for 3.66 minutes with 19 responses on average. Responses and usage duration on the next day were positively associated with SOL (responses: β = 0.05, p = .005; usage: β = 0.01, p = .03) and WASO (responses: β = 0.04, p = .03; usage: β = 0.01, p = .04) from the previous night. Daytime usage showed no significant correlation with sleep parameters that night, and nighttime use was also not associated with poor sleep that night.

#### Prediction of daily adherence to the intervention.

Good daily adherence was characterized by completing at least 1.73 sessions, spending 8.17 minutes with the chatbot, or providing 35.34 responses in a day. Table 4 shows that the LR (AUC = 0.57, p = .02), SVM (AUC = 0.56, p = .04), and GB (AUC = 0.61, p < .001) models significantly predicted good adherence based on session completion (12%, n = 116/972). The GB model (AUC = 0.74, p < .001) significantly predicted adherence based on usage duration (12%, n = 117/972). The RF (AUC = 0.58, p = .01) and GB (AUC = 0.66, p < .001) significantly predicted good adherence based on number of responses (10%, n = 101/972).

**Table 4:** Prediction of daily adherence to the intervention using machine learning with participant baseline characteristics and diary-derived sleep variables from the previous night with stratified 5-fold cross-validation.

Machine learning techniques	AUC (95% CI)	p	Sensitivity (%)	Specificity (%)
Good daily adherence in session completion (12%, n = 116/972)				
LR	0.57 (0.51, 0.62) *	.02	55.17	55.14
RF	0.52 (0.47, 0.58)	.41	50.86	50.58
SVM	0.56 (0.50, 0.62) *	.04	56.03	56.07
GB	0.61 (0.58, 0.64) ***	<.001	68.46	55.72
Good daily adherence in usage duration (12%, n = 117/972)				
LR	0.48 (0.42, 0.53)	.41	47.86	47.84
RF	0.50 (0.44, 0.56)	.96	50.43	50.99
SVM	0.47 (0.41, 0.53)	.30	47.01	47.02
GB	0.74 (0.71, 0.76) ***	<.001	78.01	59.42
Good daily adherence in response volume (10%, n = 101/972)				
LR	0.55 (0.49, 0.60)	.11	52.48	52.47
RF	0.58 (0.52, 0.64) *	.01	56.44	56.83
SVM	0.55 (0.49, 0.60)	.11	52.48	52.70
GB	0.66 (0.63, 0.68) ***	<.001	61.42	64.18

* < .05, ** < .01, *** < .001; LR: Logistic Regression with elastic-net penalty; SVM: Support Vector Machine; RF: Random Forest; GB: Gradient Boosting.

Fig 1 displays SHAP summary plots for the gradient boosting models used to predict daily adherence. For all adherence outcomes, the number of responses on the first day, along with WASO and SOL from the previous night, emerged as the most influential predictors. Notably, a higher number of responses on the first day and elevated WASO and SOL in the previous night were linked to increased predicted adherence.

## Discussion

This study is among the few to explore predictors of adherence within the context of dCBT-I [16]. The results revealed that higher baseline insomnia and depressive symptoms were associated with lower overall adherence. In particular, depressive symptoms were the strongest predictor of non-adherence. Motivational deficits associated with depression may reduce engagement with dCBT-I, and cognitive difficulties can make the intervention seem more challenging to use. Additional booster sessions targeting mood issues could benefit high-risk users with prominent sleep and mood symptoms [24,25]. Conversely, users who have a longer usage duration and provide more responses on the first day may reflect a strong initial commitment to the intervention, leading to better overall adherence. The ML analysis showed proof-of-concept performance in detecting users’ adherence. By utilizing predictive models, therapists can proactively adjust treatment strategies, for example, offering hybrid human-AI delivery to enhance patient adherence. The integration of ML may support the development of stepped-care models that customize interventions based on individual adherence and treatment response [19]. For instance, potential dropouts from fully automated digital interventions can be transferred to guided formats with human support. Such personalized approaches are crucial for increasing engagement in CBT-I, improving adherence and outcomes for individuals with insomnia.

To the best of our knowledge, no study has specifically investigated daily usage and adherence patterns in dCBT-I. Due to the variability of insomnia symptoms, participants would not interact with the chatbot on a daily basis. Thus, beyond assessing overall user behaviors, this study analyzed logging data from user responses and sleep diary entries to explore the relationship between daily sleep parameters and user behaviors. We found that the number of responses and usage duration on the following day were positively associated with SOL and WASO from the previous night. Users may be more inclined to engage with the chatbot after experiencing poor sleep, probably driven by a desire to improve their sleep quality. However, no significant correlations were found between daytime usage and sleep parameters for that specific night. While using digital devices emitting blue light before bedtime can affect sleep [26], our study indicates that nighttime use of the intervention was not associated with poor sleep on that night. While it is advisable to avoid using the chatbot before bedtime, some users might resort to it when they encounter sleep difficulties. On average, participants interacted with the chatbot just twice at night during the program, showing that the advice to limit nighttime use was useful. ML was employed to predict next-day adherence, but its performance did not match that of predicting overall adherence. Enhancing the prediction of next-day adherence may require more advanced models, larger and richer datasets.

The study reveals the challenges of adherence in delivering effective digital interventions, even when using a conversational agent. High attrition and low adherence remain pertinent issues, as highlighted by a previous systematic review [15]. The chatbot-delivered dCBT-I aimed to enhance adherence through increased interactivity with a two-way, intelligent conversational agent. However, the current study found that only 19% of the participants included completed the entire intervention. While these findings are comparable to the completion rates of 0.5-28.6% reported in prior studies [15], it still reflects the difficulty in maintaining user engagement in real-world digital interventions. Nevertheless, findings indicate that the chatbot improved SE for participants who did or did not have good adherence to the intervention, suggesting benefits even for those who only partially engaged with the intervention [24]. It is possible that those who discontinue treatment early did so because of an early and favorable response to treatment. The results highlight the potential of the chatbot-delivered dCBT-I as an accessible and effective intervention for improving sleep in a real-world setting. Moreover, those with good adherence experienced a greater reduction in WASO, indicating a potential link between adherence and the effectiveness of dCBT-I. Future research should explore whether those who discontinue treatment early are still able to achieve clinically meaningful improvements.

The study has several limitations that should be considered. First, the relatively small sample size, particularly the number of participants who completed the baseline questionnaire and the full intervention, may limit the statistical power and the ability to detect more nuanced relationships. Potential selection bias and concerns regarding the representativeness of the sample could further restrict the generalizability of our findings. Second, the reliance on self-reported data introduces possible recall biases. The use of objective sleep measures, such as actigraphy, could provide a more comprehensive assessment of the intervention effect on sleep parameters. Third, the study did not compare a wide range of machine learning algorithms, model parameters, or feature sets to optimize results. We used simpler models for this proof-of-concept, so the predictive performance may be underestimated. Fourth, the focus on a chatbot-delivered dCBT-I in a specific cultural context may limit the generalizability of the findings to other populations. Comparative studies evaluating the chatbot in diverse cultural and demographic settings would be valuable. Inclusion of participants with psychiatric comorbidities could enhance the study’s relevance and generalizability across broader clinical populations.

Future research should explore the potential moderating factors that may influence the adherence and effectiveness of chatbot-delivered dCBT-I. Exploring the specific features, functionalities, and design elements of the chatbot that enhance user engagement and adherence would be valuable for creating more effective chatbot-delivered interventions. For instance, incorporating a virtual therapist into the conversational agent, complete with designed animations and additional visual presentations, could enable more interactive and personalized interventions.

## Conclusion

Challenges related to user adherence persist, with low completion rates indicating the need for further enhancements in digital mental health interventions. This study utilizes ML techniques to predict adherence to dCBT-I in a real-world setting, enhancing the ecological validity of the findings. The integration of ML to identify user engagement patterns offers promising avenues for personalizing treatment and optimizing adherence strategies. Leveraging predictive analytics will be crucial for improving the reach of dCBT-I, ultimately contributing to better sleep health outcomes across populations.

## Supporting information

S1 Fig The intervention service and questionnaire administration. The intervention service and questionnaire administration were automated and conducted through online platforms.
(TIFF)

S2 Fig A sleep diary entry. The chatbot automatically determined sleep efficiency using the daily sleep diary, which included sleep-wake patterns such as total sleep time, total time in bed, wake time after sleep onset, and sleep onset latency.
(TIFF)

S3 Fig An example of the chatbot-user interactions on the topic of sleep psychoeducation. The intervention was provided through multimedia formats.
(TIFF)

S4 Fig An example of a user asking the chatbot a question about the sleep diary. The chatbot facilitated the Q&A feature, allowing users to inquire about the intervention materials.
(TIFF)
