# Supplementary figures and images for: Predicting adherence to fully-automated, chatbot-delivered digital cognitive behavioral therapy for insomnia (dCBT-I) using machine learning: A pilot real-world study
Source: PLOS Digit Health. 2026 Jan 2;5(1):e0001170. doi: 10.1371/journal.pdig.0001170 (PMC12758786; doi:10.1371/journal.pdig.0001170)

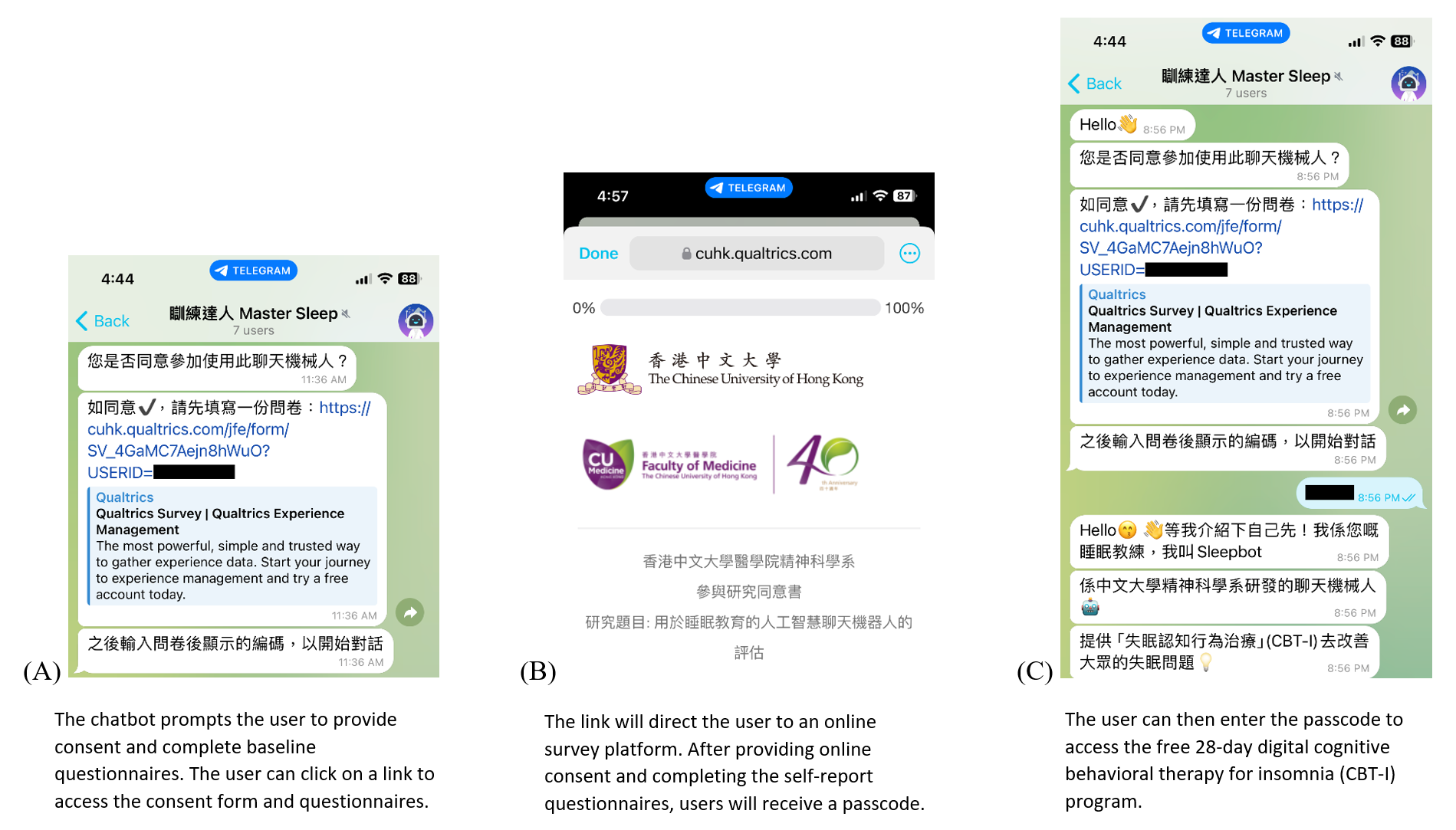

Supplement: S1 Fig — The intervention service and questionnaire administration were automated and conducted through online platforms. (TIFF) [file pdig.0001170.s001.tiff]

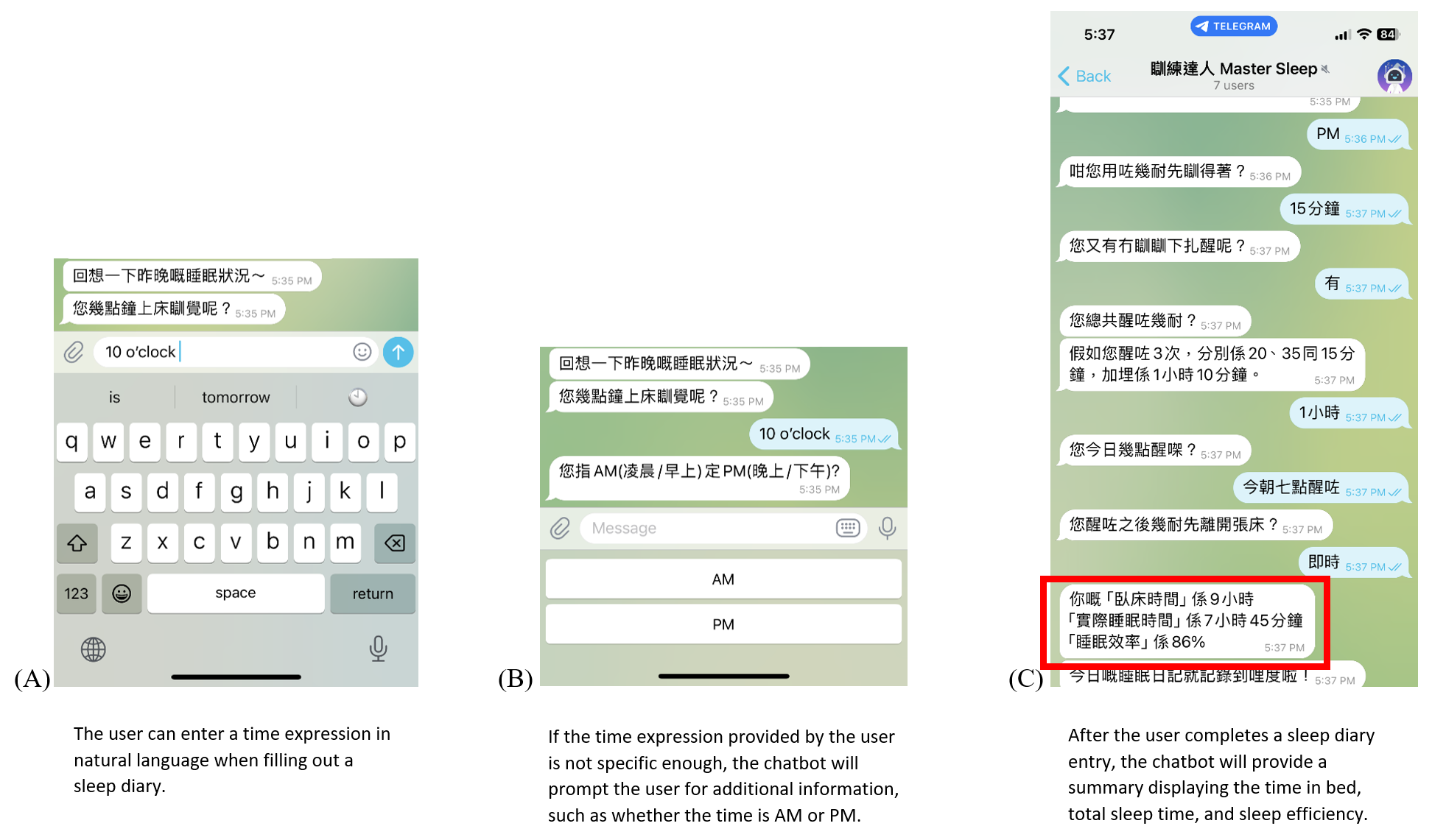

Supplement: S2 Fig — The chatbot automatically determined sleep efficiency using the daily sleep diary, which included sleep-wake patterns such as total sleep time, total time in bed, wake time after sleep onset, and sleep onset latency. (TIFF) [file pdig.0001170.s002.tiff]

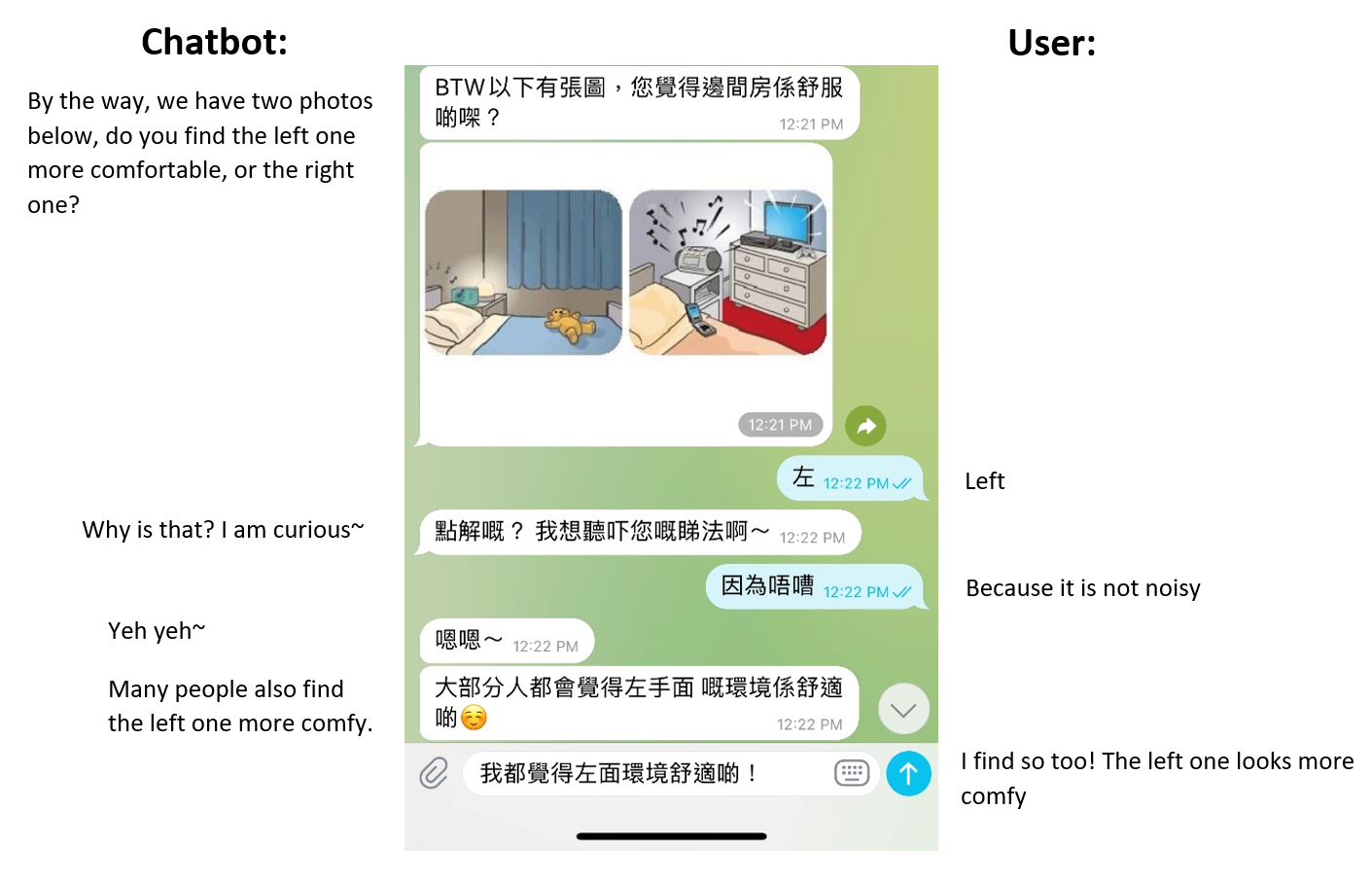

Supplement: S3 Fig — The intervention was provided through multimedia formats. (TIFF) [file pdig.0001170.s003.tiff]

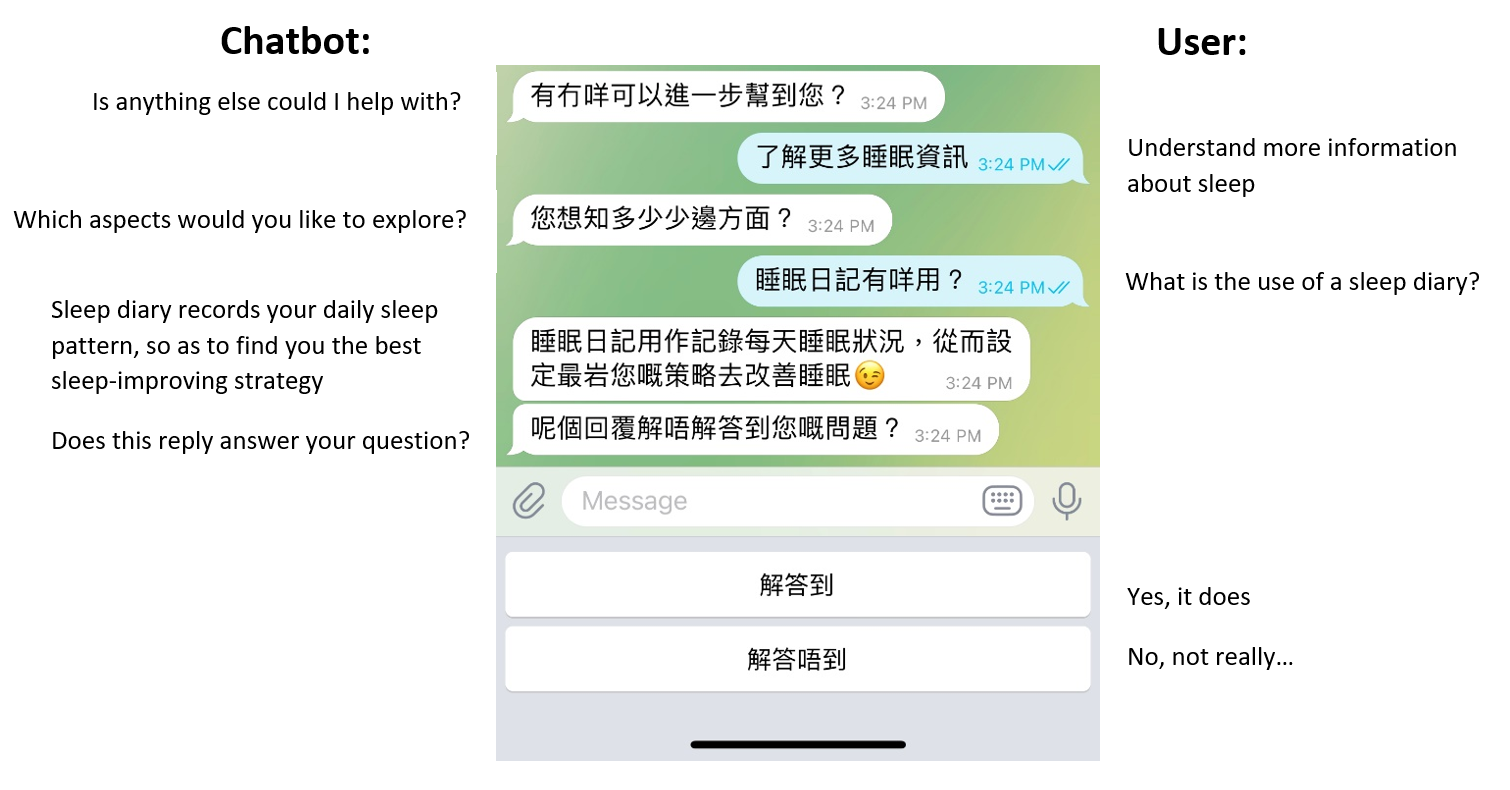

Supplement: S4 Fig — The chatbot facilitated the Q&A feature, allowing users to inquire about the intervention materials. (TIFF) [file pdig.0001170.s004.tiff]
